# A SAR and QSAR Study of New Artemisinin Compounds with Antimalarial Activity

**DOI:** 10.3390/molecules19010367

**Published:** 2013-12-30

**Authors:** Cleydson Breno R. Santos, Josinete B. Vieira, Cleison C. Lobato, Lorane I. S. Hage-Melim, Raimundo N. P. Souto, Clarissa S. Lima, Elizabeth V. M. Costa, Davi S. B. Brasil, Williams Jorge C. Macêdo, José Carlos T. Carvalho

**Affiliations:** 1 Laboratory of Modeling and Computational Chemistry, Federal University of Amapá, Macapá 68902-280, Amapá, Amazon, Brazil; E-Mails: jnetbio.unifap2011.ap@gmail.com (J.B.V.); cleyson.cl@gmail.com (C.C.L.); lorane@unifap.br (L.I.S.H.-M.); williamsmacedo@yahoo.com.br (W.J.C.M.); 2 Postgraduate Program in Biotechnology and Biodiversity-Network BIONORTE, Macapá 68902-280, Amapá, Amazon, Brazil; E-Mails: rnpsouto@unifap.br (R.N.P.S.); farmacos@unifap.br (J.C.T.C.); 3 Laboratory of Drug Research, School of Pharmaceutical Sciences, Federal University of Amapá, Macapá 68902-280, Amapá, Amazon, Brazil; E-Mails: lima.clarissa@gmail.com (C.S.L.); elizabethviana@unifap.br (E.V.M.C.); 4 Institute of Technology, Federal University of Pará, Av. Augusto Corrêa, 01, Belém 66075-900, Pará, Amazon, Brazil; E-Mail: davibb@ufpa.br

**Keywords:** artemisinin, antimalarial activity, HF/6-31G**, molecular docking, MEPs, SAR, QSAR

## Abstract

The Hartree-Fock method and the 6-31G** basis set were employed to calculate the molecular properties of artemisinin and 20 derivatives with antimalarial activity. Maps of molecular electrostatic potential (MEPs) and molecular docking were used to investigate the interaction between ligands and the receptor (heme). Principal component analysis and hierarchical cluster analysis were employed to select the most important descriptors related to activity. The correlation between biological activity and molecular properties was obtained using the partial least squares and principal component regression methods. The regression PLS and PCR models built in this study were also used to predict the antimalarial activity of 30 new artemisinin compounds with unknown activity. The models obtained showed not only statistical significance but also predictive ability. The significant molecular descriptors related to the compounds with antimalarial activity were the hydration energy (HE), the charge on the O11 oxygen atom (QO11), the torsion angle O1-O2-Fe-N2 (D2) and the maximum rate of R/Sanderson Electronegativity (RTe^+^). These variables led to a physical and structural explanation of the molecular properties that should be selected for when designing new ligands to be used as antimalarial agents.

## 1. Introduction

Malaria is a very serious infectious disease caused by protozoans of the genus *Plasmodium* and is transmitted through the bite of infected female *Anopheles* mosquitoes. Every year, over one million people die from malaria, especially in tropical and subtropical areas. Most of the deaths are attributed to the parasite species *Plasmodium falciparum*. Many drugs have been investigated for their efficacy in the treatment of the disease, but strains of *P. falciparum* resistant to some of these drugs have appeared. Hence, the discovery of new classes of more potent compounds to treat the disease is necessary [1,2,3,4,5,6]. Artemisinin (qinghaosu) has been used in traditional Chinese medicine to treat disease for more than two million years. The medicine is extracted from the plant *Artemisia annua* L. and is used to combat 52 species of diseases in the People’s Republic of China [7]. Artemisinin has a unique structure with a stable endoperoxide lactone (1, 2, 13-trioxane) that is totally different from previous antimalarials in its structure and mode of action. Artemisinin is remarkably effective against *Plasmodium falciparum* and cerebral malaria [8]. Currently, semi-synthetic artemisinin derivatives play an important role in the treatment of *P*. *falciparum* malaria [9,10,11]. Although the true mechanism of their biological activity against malaria has not been completely elucidated, various studies suggest that the trioxane ring is essential for antimalarial activity due to the properties displayed by the endoperoxide linkage. The literature also suggests that free heme could be the target of artemisinin in biological systems and that Fe^2+^ interacts with the peroxide when artemisinin reacts with heme [12,13,14,15]. Artemisinin and its derivatives induce a rapid reduction in the number of parasites when compared with other known drugs. Consequently, they are of particular interest for severe cases of malaria. The initial decline in the number of parasites is also beneficial for combination therapies. Therefore, there is an enormous interest in the mechanism of action, chemistry and drug development of this new class of antimalarials. The endoperoxide group is essential for the antimalarial activity and is mediated by activated oxygen (superoxide, H_2_O_2_ and/or hydroxyl radicals) or carbon free radicals [16,17,18,19].

In the evolution of computational chemistry, the use of molecular modeling (MM) has been one of the most important advances in the design and discovery of new drugs. Currently, MM is an indispensable tool in not only the process of drug discovery but also the optimization of existing prototypes and the rational design of drug candidates [20,21,22,23]. According to IUPAC, MM is the investigation of molecular structures and properties using computational chemistry and graphical visualization techniques to provide a three-dimensional representation of the molecule under a given set of circumstances [21]. The nature of the molecular properties used and the extent to which they describe the structural features of molecules can be related to biological activity, which is an important part of any Structure-Activity Relationship (SAR) or Quantitative Structure-Activity Relationship (QSAR) study. QSAR studies use chemometric methods to describe how a given biological activity or a physicochemical property varies as a function of the molecular descriptors describing the chemical structure of the molecule. Thus, it is possible to replace costly biological tests or experiments using a given physicochemical property (especially those involving hazardous and toxically risky materials or unstable compounds) with calculated descriptors that can, in turn, be used to predict the responses of interest for new compounds [24]. Recently, Cristino *et al*. studied nineteen 10-substituted deoxoartemisinin derivatives and artemisinin with activity against D-6 strains of malarial *falciparum* in Sierra Leone. They used chemometric modeling to reduce dimensionality and determine which subset of descriptors are responsible for the classification between more active (MA) and less active (LA) artemisinins. A predictive study was performed with a new set of eight artemisinins using chemometric methods, and five of them were predicted to be active against D-6 strains of *falciparum* malaria [25].

**Figure 1:**
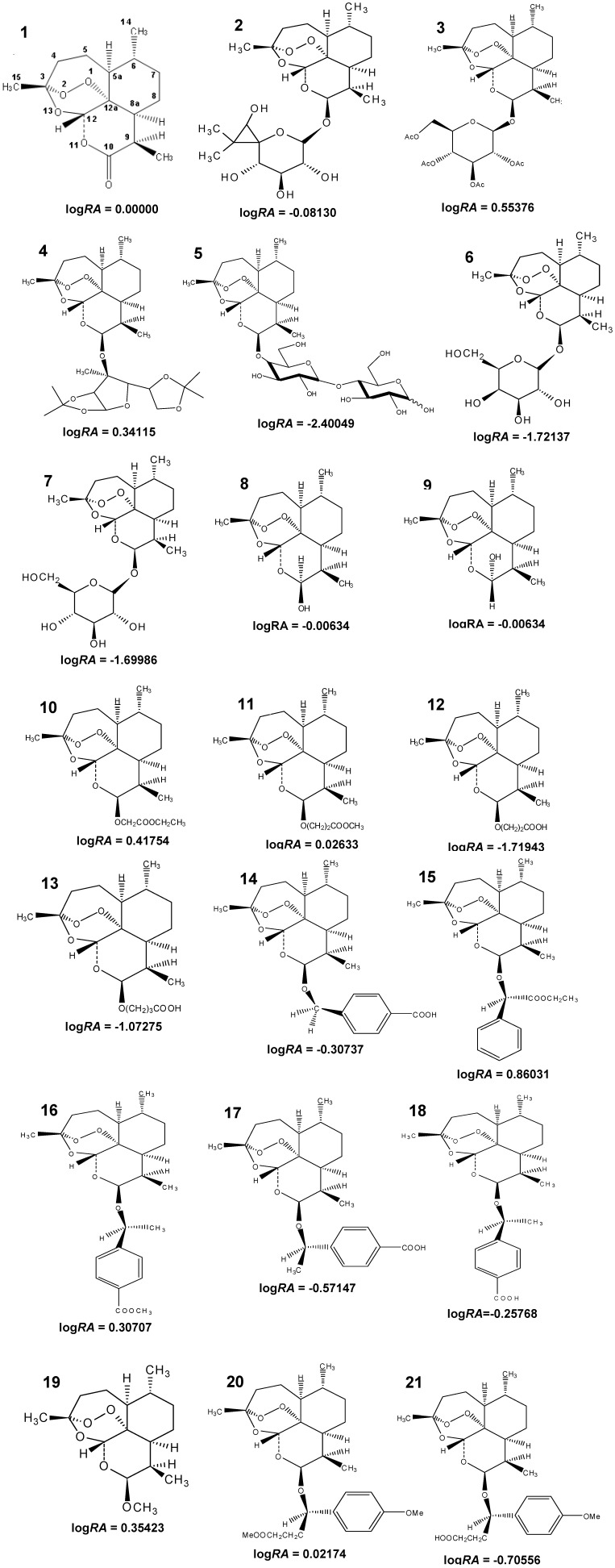
Structure and biological activity of artemisinin derivatives.

In this paper, a SAR and QSAR study of artemisinin and 20 derivatives (see Figure 1) with different antimalarial activities, tested *in vitro* against *P. falciparum* (W-2), was performed*.* Initially, the structures were modeled, and many different molecular descriptors were computed. Maps of the molecular electrostatic potential (MEP) and molecular docking were employed to better understand the correlation between structure and activity and the interaction between the ligands (artemisinin and derivatives) and the receptor (heme). Multivariate analysis methods were used to deal with the large number of descriptors and generate a predictive model [26]. Principal Component Analysis (PCA) and Hierarchical Cluster Analysis (HCA) were employed to choose the molecular descriptors that are most related to the biological property investigated. Then, a QSAR model was elaborated through the Principal Component Regression (PCR) and Partial Least Square (PLS) methods that were used to perform predictions of 30 new artemisinin compounds with unknown antimalarial activity and to aid in future studies searching for other new antimalarial drugs [27,28,29].

## 2. Results and Discussion

### 2.1. Optimization of the Geometry of Artemisinin in Different Methods and Basis Sets

In all three basis sets (HF/6-31G, HF/6-31G*, HF/6-31G**), the Hartree-Fock method describes all structural parameters very well in terms of magnitude and sign when compared to the experimental values (see Table 1). This is in contrast to the AM1, PM3, ZINDO and DFT (B3LYP/3-21G, B3LYP/3-21G*, B3LYP/3-21G**) methods, in which there is not good agreement between the experimental and theoretical values for the torsion angles, especially the angle formed by atoms C3O13C12C12a, with deviations <−13.900° (AM1), <−22.489° (PM3), <−7.880° (ZINDO), >0.020° (HF/6-31G), >2.132° (HF/6-31G*), >2.100° (HF/6-31G**) > −3.759° (B3LYP/3-21G), >−3.760° (B3LYP/3-21G*) and >−3.780° (B3LYP/3-21G**) and standard deviations of 4.776, 8.388, 4.372, 1.663, 2.484, 1.762, 1.915, 1.855 and 1.987, respectively. By comparing these methods with the HF method, we find that the HF/6-31G and HF/6-31G** basis sets have low standard deviations in relation to the semiempirical and DFT methods. The variation was ±0.099 between HF/6-31G and HF/6-31G**.

Table 1 shows that the HF/6-31G, HF/6-31G*, HF/6-31G** basis sets show excellent results for bond length compared to the experimental data. The 6-31G basis set described the bond angles well, with values close to the experimental results. However, the minimum bases (6-31G and 3-21G) have several deficiencies; thus, a polarization function was included to improve upon these bases (*i.e*., *p* orbitals represented by *). These orbitals follow restricted functions that are centered at the nuclei. However, the atomic orbitals become distorted or polarized when a molecule is formed. Therefore, one must consider the possibility of non-uniform displacement of electric charges outside of the atomic nucleus, *i.e.*, polarization. Thus, it is possible to obtain a better description of the charges and deformations of atomic orbitals within a molecule. A mode of polarization can be considered by introducing functions for which the values of *l* (quantum number of the orbital angular momentum) are larger than those of the fundamental state of a given atom. For these types, the basis set names denote the polarization functions. Thus, 6-31G* refers to basis set 6-31G with a polarization function for heavy atoms (*i.e*., atoms other than hydrogen), and 6-31G** refers to the inclusion of a polarization function for hydrogen and helium atoms [30]. When basis sets with polarization functions are used in calculations involving anions, good results are not obtained due to the electronic cloud of anionic systems, which tend to expand. Thus, appropriate diffuse functions must be included because they allow for a greater orbital occupancy in a given region of space. Diffuse functions are important in the calculations of transition metals because metal atoms have “*d*” orbitals, which tend to be diffuse. It then becomes necessary to include diffuse functions in the basis function associated with the configuration of a neutral metal atom to obtain a better description of the metal complex. The 6-31G** basis is particularly useful in the case of hydrogen bonds [30,31,32,33,34].

This study highlighted that the HF/6-31G** basis set, which is closer to the experimental results and shows good performance in the description when comparing the C3O13C12 and C12aO1O2 bond angles. The torsion angles or dihedral angle also showed good agreement with the experimental values reported in the literature, showing that with the 6-31G** basis set, the torsion angles O1O2C3O13 and C13C12C12aO1 are closer to the crystallographic data. Artemisinin derivatives with antimalarial activity against *Plasmodium falciparum*, which is resistant to mefloquine, were studied using quantum chemical methods (HF/6-31G*) and the partial least-squares (PLS) method. Three main components explained 89.55% of the total variance, with *Q*^2^ = 0.83 and *R*^2^ = 0.92. From a set of 10 proposed artemisinin derivatives (artemisinin derivatives with unknown antimalarial activity against *Plasmodium falciparum*), a novel compound was produced with superior antimalarial activity compared to the compounds previously described in the literature [35]. Cardoso *et al*. [36] used HF/3–21G** *ab initio* and PLS methods to design new artemisinin derivatives with activity against *P*. *falciparum* malaria. The PLS method was used to build a multivariate regression model, which led to new artemisinin derivatives with unknown antimalarial activity. Additionally, MEP maps for the studied and proposed compounds were built and evaluated to identify common features in active molecules.

Cardoso *et al*. [37] studied artemisinin and some of its derivatives with activity against D-6 strains of *Plasmodium falciparum* using the HF/3-21G method. To verify the reliability of the geometry obtained, Cardoso *et al*. compared the structural parameters of the artemisinin trioxane ring with theoretical and experimental values from the literature. Ferreira *et al.* [16] studied artemisinin and 18 derivatives with antimalarial activity against W-2 strains of *Plasmodium falciparum* through quantum chemistry and multivariate analysis. The geometry optimization of structures was performed using the Hartree-Fock method and the 3-21G** basis set. Recently, Santos *et al*. [38] validated the HF/6-31G** computational methods applied in the molecular modeling of artemisinin, proposing a combination of chemical quantum methods and statistical analysis to study geometrical parameters of artemisinin in the region of the 1, 2, 13-trioxane endoperoxide ring. In determining the most stable structures of the studied compounds as well as the molecular properties, the Hartree-Fock method with the 6-31G** valence basis set separately has been used instead of semiempirical approaches such as AM1, PM3 and ZINDO, due to the number of relatively small compounds.

**Table 1:** Theoretical and experimental parameters of the 1, 2, 13-trioxane ring in artemisinin.

Parameters ^[a]^	Semiempirical			Hartree-Fock/HF			DFT/B3LYP			Experimental ^[f]^
	AM1 ^[b, c]^	PM3 ^[b, c]^	ZINDO ^[b, c]^	6-31G ^[b, c]^	6-31G* ^[b, c]^	6-31G** ^[d]^	3-21G ^[e]^	3-21G* ^[e]^	3-21G**^[e]^	
Bond Length (Å)										
O1O2	1.288	1.544	1.237	1.447	1.391	1.390	1.524	1.524	1.524	1.469
O2C3	1.447	1.403	1.400	1.435	1.393	1.396	1.455	1.455	1.454	1.416
C3O13	1.427	1.428	1.396	1.435	1.388	1.408	1.473	1.473	1.472	1.445
O13C12	1.416	1.403	1.392	1.403	1.400	1.376	1.430	1.430	1.430	1.379
C12C12a	1.537	1.555	1.513	1.533	1.533	1.532	1.535	1.535	1.535	1.523
C12aO1	1.468	1.426	1.416	1.469	1.429	1.429	1.504	1.504	1.504	1.461
Bond Angle (°)										
O1O2C3	112.530	110.340	114.310	108.800	106.100	109.460	105.590	105.590	105.480	108.100
O2C3O13	103.600	104.810	105.370	106.760	110.800	107.800	108.220	108.220	108.250	106.600
C3O13C12	115.480	116.010	115.843	117.300	112.800	115.300	113.200	113.200	113.200	114.200
O13C12C12a	113.510	115.200	113.270	112.280	108.700	112.300	113.300	113.300	113.230	114.500
C12C12aO1	111.070	113.180	107.290	110.910	110.500	110.545	112.410	112.410	112.470	110.700
C12aO1O2	113.740	112.290	118.380	113.240	112.700	112.700	109.620	109.620	109.590	111.200
Torsion Angle (°)										
O1O2C3O13	−77.800	−73.310	−70.403	−71.840	−73.369	−73.400	−76.610	−76.610	−76.740	−75.500
O2C3O13C12	42.070	52.700	36.370	33.390	31.034	31.100	33.750	33.750	33.720	36.000
C3O13C12C12a	11.400	2.811	17.420	25.320	27.432	27.400	29.059	29.060	29.080	25.300
O13C12C12aO1	−41.770	−40.510	−46.610	−49.410	−50.100	−50.143	−52.190	−52.190	−52.030	−51.300
C12C12aO1O2	12.050	19.940	18.110	12.510	10.900	10.924	9.060	9.600	9.340	12.700
C12aO1O2C3	47.050	35.630	40.130	46.700	48.700	48.674	51.060	51.060	51.320	47.800
Standard Deviation	4.776	8.388	4.372	1.663	2.484	1.762	1.915	1.855	1.987	-

^[a]^: The atoms are numbered according to compound 1 in Figure 1; ^[b]^ Ref. [36]; ^[c]^ Ref. [37]; ^[d]^ Valence basis set separately validated to calculate the molecular properties; ^[e]^ Ref. [38]; ^[f]^: Ref. [39].

### 2.2. Molecular Docking

**Figure 2:**
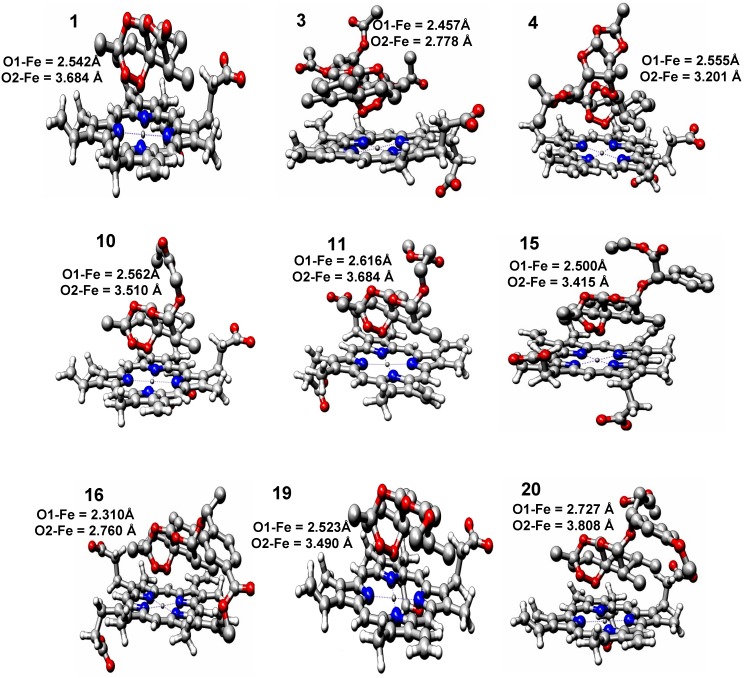
Heme-artemisinin interactions of the most active compounds (**1**, **3**, **4**, **10**, **11**, **15**, **16**, **19** and **20**).

Docking calculations showed that the entire ligand molecule is placed parallel to the plane of the porphyrin ring of heme, and the polar part of the ligand, which contains the peroxide bond, is directed toward the polar part of the heme system containing Fe^2+^. This interaction is visualized in Figure 2 for most active compounds (1, 3, 4, 10, 11, 15, 16, 19 and 20). These orientations were assumed to be the most favorable and therefore to represent the real system under investigation, given that they were chosen based on the lowest free-energy of binding (interaction energy). For the compounds in the studied set, the values of *d*(Fe–O1) ranged from 2.310 to 2.727 Å; however, this interval for the *d*(Fe–O2) distances ranged from 2.760 to 3.808 Å. The *d*(Fe–O13) distances ranged from 4.811 to 5.434, and the *d*(Fe–O11) distances ranged from 4.897 to 5.525, as shown in Table 2.

**Table 2:** Parameters calculated by molecular docking of heme-artemisinin and most active derivatives.

Compounds	E_Complex_ (Kcal mol^−1^)	Fe–O1 Distance (Å)	Fe–O2 Distance (Å)	Fe–O13 Distance (Å)	Fe–O11 Distance (Å)	log*RA*
1	−6.06	2.542	3.684	5.153	5.525	0.00000
3	−5.09	2.457	2.778	4.811	5.202	0.55376
4	−6.54	2.555	3.201	4.982	5.448	0.34115
10	−5.27	2.562	3.510	5.184	5.404	0.41754
11	−5.37	2.616	3.684	5.300	5.364	0.02633
15	−4.70	2.500	3.415	5.127	5.351	0.86031
16	−5.53	2.310	2.760	4.874	4.897	0.30707
19	−5.99	2.523	3.490	5.158	5.357	0.35423
20	−5.03	2.727	3.808	5.434	5.475	0.02174
E_Complex_		0.06551	0.01761	0.19250	−0.20162	0.38917
Fe–O1			0.84202	0.85273	0.83598	−0.44984
Fe–O2				0.94792	0.81259	−0.48039
Fe–O13					0.65135	−0.48864
Fe–O11						−0.27755

For artemisinin (1), the *d*(Fe–O1) calculated distance was 2.542 Å, which is very close to the value reported (2.7 Å) in other theoretical studies [40,41]. There is a clear trend involving interatomic separation between Fe^2+^ and the oxygen atom in the trioxane ring because the distances are shorter for the O1 atom than for the O2 atom. This result reinforces the idea that the O1 atom from artemisinin preferentially binds to the Fe^2+^ from heme instead of the O2 atom.

Compounds **4**, **10**, **11** and **20** have higher activity than artemisinin and also higher values of *d*(Fe–O1). They have a large substituent that certainly causes repulsion due to steric effects, which prevents them from binding closer to the heme. Compounds **5** and **6** were designed to increase lipophilicity because it was observed that higher lipophilicity of artemisinin correlates with greater biological activity. Compounds **15**, **16** and **20** present large substituent groups on the α-methylene carbon (*C) that substantially increase the antimalarial activity of the compounds due to electronic and steric effects, respectively. Compound **3** demonstrated that the sugar-containing dihydroartemisinin acetylation derivatives have similar or better activities than artemisinin. However, the deacetylation of sugars reduces the antimalarial activity considerably.

The interaction energy for the ligand/receptor complex showed good linear correlation with activity (*r* = 0.389177) and ranged from −6.54 to −5.03 kcal·mol^−1^ when compared with Fe–O1, Fe–O2, Fe–O13 and Fe–O11 distances (Å) (Table 2). In fact, even though some orientations were associated with the lowest interaction energy, they seemed to have strong activity against malaria because they presented the endoperoxide bond away from Fe^2+^. Currently, the most accepted mechanisms of antimalarial action involve the formation of a complex between heme and artemisinin derivatives in which the iron of heme interacts with O1 of the endoperoxide. Moreover, substituent and conformation effects may affect the charge distribution at the oxygen and even the Fe–O1 bond [35]. An increase in the polar area of artemisinin increases the polar interactions between heme, the ligand and the globin.

### 2.3. Molecular Electrostatic Potential Maps

To identify key characteristics of compounds derived from artemisinin, maps of molecular electrostatic potential (MEPs) were evaluated and used for qualitative comparisons in the region of the 1, 2, 13-trioxane ring of artemisinin and its derivatives. The geometrical form of the potential in the region of the 1, 2, 13-trioxane ring is similar for all active compounds and is characterized by negative electrostatic potential (red region) according to the literature [42]. 

**Figure 3:**
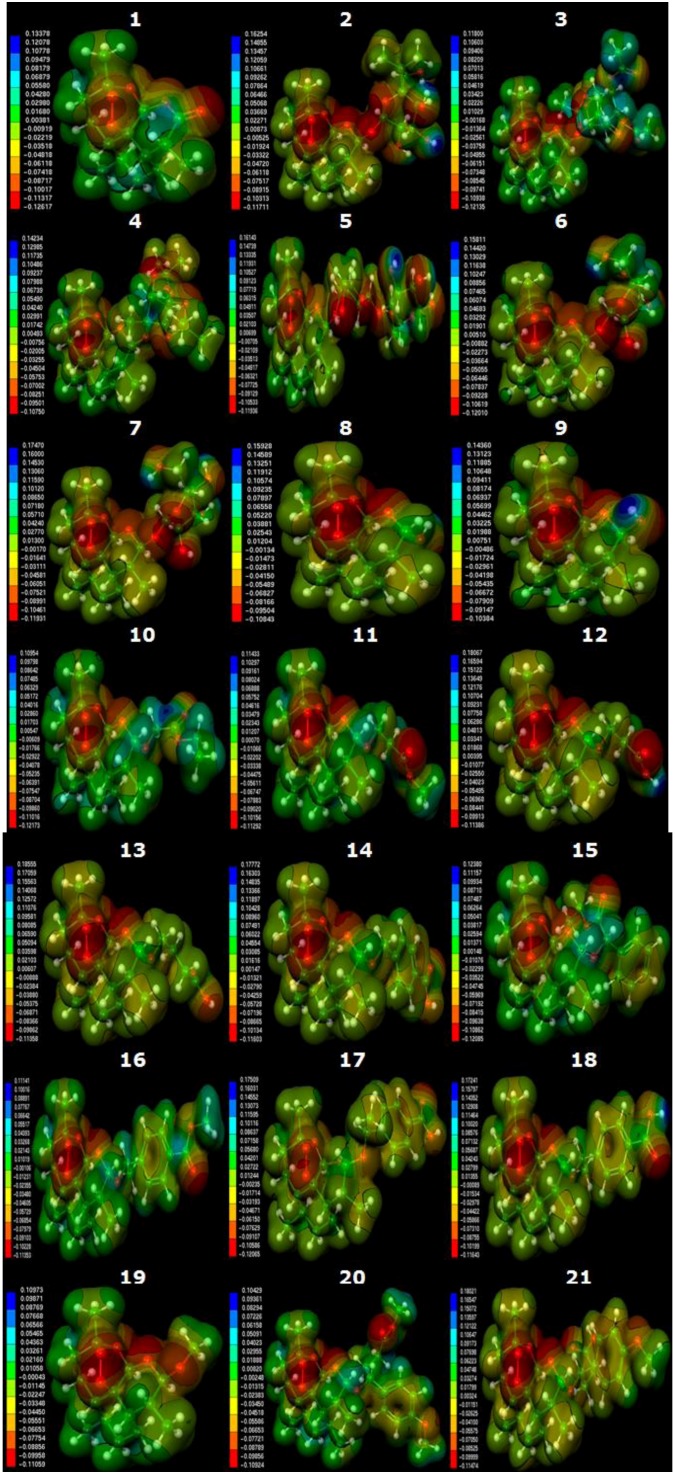
Molecular electrostatic potential maps of the studied artemisinin derivatives with antimalarial activity against *Plasmodium falciparum* (W-2 clone).

The MEP visualization is shown in Figure 3. Compounds **2**–**21** have a region of negative potential near the trioxane ring, similar to the MEP of artemisinin (compound 1), which has an electrostatic potential maximum of 0.13378 u.a. (blue region) and a minimum of −0.12617 u.a. (red region). The maximum positive MEP (blue region) varied from 0.14234 u.a. 0.10429 u.a. for active compounds, while less active compounds ranged from 0.18555 u.a. to 0.14360 u.a. The values corresponding to the minimum negative electrostatic potential (red region) for the most active compounds ranged from −0.10750 u.a. to −0.12617 u.a., presenting potential values close to those of artemisinin. The minimum negative electrostatic potential (red region) for less active compounds ranged from −0.10384 u.a. to −0.12065 u.a., which are higher than those of artemisinin. 

The region of negative electrostatic potential is due to the binding of the endoperoxide (C-O-O-C), which is the most notable feature of MEP. The distribution of the electron density around the trioxane ring is thought to be responsible for activity against malaria, a belief supported by the fact that the complexation of artemisinin with heme involves an interaction between the peroxide bond, the most negatively charged zone on the ligand, and Fe^2+^, the most positively charged zone on heme (the receptor molecule) [15,43]. 

The presence of a negative surface close to the trioxane ring suggests that these compounds have a reactive site for electrophilic attack and must possess antimalarial potency; consequently they are being investigated. Thus, in the case of an electrophilic attack of the iron of heme against an electronegative zone, there is a preference for it to occur through the endoperoxide linkage. By analyzing *MEP* maps, the selection of inactive compounds can be avoided.

### 2.4. PCA Results

The PCA results showed that the most important descriptors were the following: the hydration energy (HE), charge on the oxygen atom O11 (QO11), torsion angle D2 (O2–O1–Fe–N2) and the maximum rate of R/Sanderson electronegativity (RTe^+^). The hydration energy is the energy released when water molecules are separated from each other and are attracted by solute molecules or ions. Hydration energy comprises solvent-solvent and solute-solvent interactions [44]. The charge on the O11 atom (QO11) is a measure of the force with which a particle can electrostatically interact with another particle [45]. O RTe^+^ is a GETAWAY (geometry, topology and set of atomic weights) type descriptor associated with the form, symmetry size and molecular distribution of the atom [46,47]. The torsion angle D2 (O2–O1–Fe–N2) is of great importance in our study; according to the proposal of Jefford and colleagues, the iron of heme attacks artemisinin at O1 and generates a free radical in position O2 after the C3-C4 bond is broken, generating a carbon radical at C4 [48]. This free radical at C4 has been suggested to be an important component of antimalarial activity [49]. Molecular docking of artemisinin and its receptor, the heme group, performed by Tonmunphean, Parasuk and Kokpol also indicated that the iron of the heme group preferentially interacts with O1 rather than O2 [41].

The values of the important descriptors of each selected compound identified via PCA as well as the values of log*RA*, relative activity (RA) and the *IC*_50_ is the 50% inhibitory concentration are shown in Table 3. The Table 3 shows the Pearson correlation matrix between the descriptors and log*RA*, and the correlation between pairs of descriptors is less than 0.70, while the correlation between the descriptors and log*RA* is less than 0.87. The descriptors selected by PCA represent the characteristics necessary to quantify the antimalarial activity of these compounds against *Plasmodium falciparum* W-2.

The results of the SAR model are presented in Table 4. The model was constructed with three main components (3 PCs). The first principal component (PC1) describes 40.8865% of the total information, the second principal component (PC2) describes 22.7045%, and the third (PC3) 11.5660%. PC1 contains 51.1081% of the original data, and the combination of the first two components (PC1 + PC2) contains 79.4887%, and all three (PC1 + PC2 + PC3) explain 93.9461% of the total information, losing only 6.0539% of the original information. The descriptors HE, D2 and QO11 contribute the most to PC1, while in PC2, the descriptor RTe^+^ is the primary contributor. The main components can be written as a linear combination of the selected descriptors. Mathematical expressions for PC1 and PC2 are shown below.

PC1 = 0.5705HE - 0.5088QO11 + 0.0925RTe^+^ + 0.6381D2 (1)

PC2 = -0.3847HE - 0.2987QO11 + 0.08731RTe^+^ - 0.0207D2 (2)

**Figure 4:**
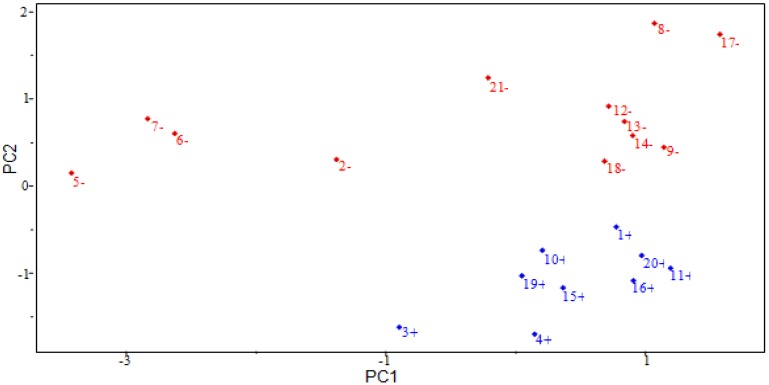
Plot of PC1–PC2 scores for artemisinin and derivatives with antimalarial activity against W-2 strains of *P. falciparum*. Positive values indicate more potent analogs, and negative values indicate less potent analogs.

Figure 4 shows the scores for the 21 compounds studied. Based on the graph, PC2 distinguishes between compounds that are more potent and less potent. The most potent compounds are located at the bottom (**1**, **3**, **4**, **10**, **11**, **15**, **16**, **19** and **20**), while the less potent compounds are located in the upper portion of the graph (**2**, **5**, **6**, **7**, **8**, **9**, **12**, **13**, **14**, **17**, **18** and **21**).

**Table 3:** Physicochemical properties selected by principal component analysis, experimental log*RA* values, *IC*_50_ and the correlation matrix.

Compounds	HE	QO11	RTe^+^	D2	log*RA*	*RA*	IC_50_ (ng/mL)
1^+^	−2.820	−0.605	0.105	120.868	0.00000	1.000000	0.6800
2^−^	−13.330	−0.516	0.127	27.480	−0.08130	0.829268	0.8200
3^+^	−4.190	−0.567	0.066	−62.834	0.55376	3.578947	0.1900
4^+^	−2.970	−0.558	0.069	94.272	0.34115	2.193548	0.3100
5^−^	−26.220	−0.558	0.079	−143.766	−2.40049	0.003977	171.0000
6^−^	−16.200	−0.547	0.123	−163.237	−1.72137	0.018994	35.6000
7^−^	−16.640	−0.517	0.135	−158.396	−1.69986	0.019959	34.0700
8^−^	−3.690	−0.631	0.187	111.395	−0.00634	0.985507	0.6900
9^−^	−5.670	−0.676	0.126	113.465	−0.00634	0.985507	0.6900
10^+^	−1.680	−0.599	0.100	13.716	0.41754	2.615385	0.2600
11^+^	−3.330	−0.662	0.080	124.375	0.02633	1.062500	0.6400
12^−^	−8.530	−0.666	0.131	105.696	−1.71943	0.019080	35.6400
13^−^	−8.210	−0.648	0.132	138.353	−1.07275	0.084577	8.0400
14^−^	−7.420	−0.651	0.131	127.415	−0.30737	0.492754	1.3800
15^+^	−2.930	−0.675	0.068	−17.600	0.86031	7.249467	0.0938
16^+^	−2.540	−0.637	0.081	100.819	0.30707	2.028035	0.3353
17^−^	−6.900	−0.754	0.150	95.974	−0.57147	0.268245	2.5350
18^−^	−6.980	−0.645	0.118	107.153	−0.25768	0.552486	1.2308
19^+^	−1.870	−0.501	0.105	114.392	0.35423	2.260638	0.3008
20^+^	−5.560	−0.641	0.082	153.908	0.02174	1.051330	0.6468
21^−^	−11.120	−0.651	0.141	10.910	−0.70556	0.196987	3.4520
EH		−0.329	−0.156	0.694	0.860	-	-
QO11			−0.203	−0.509	−0.127	-	-
RTe+				0.128	−0.333	-	-
D2					0.485	-	-

**Table 4:** Principal component analysis of the SAR model and contribution of selected descriptors based on step multivariate analysis.

Parameters	Main Component		
	PC1	PC2	PC3
Variance (%)	40.8865	22.7045	11.5660
Cumulative Variance (%)	51.1081	79.4887	93.9461
Molecular Descriptors		**Contribution**	
		**PC1**	**PC2**
EH		0.5705	−0.3847
QO11		−0.5088	−0.2987
RTe^+^		0.0925	0.8731
D2		0.6381	−0.0207

**Figure 5:**
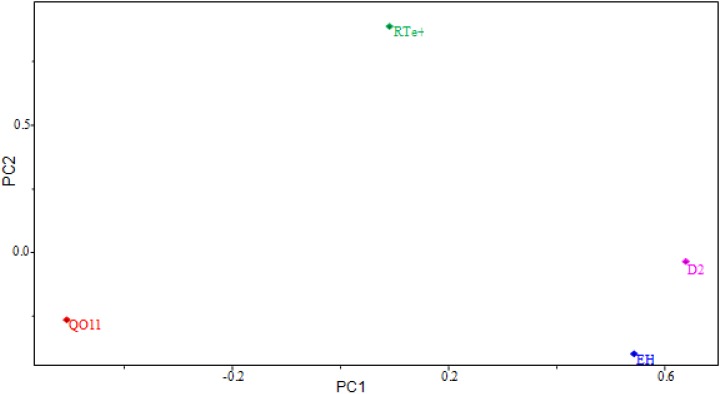
Plot of the PC1–PC2 loadings with the four descriptors selected to build the PLS and PCR models of artemisinin and derivatives with biological activity against W-2 strains of *P. falciparum*.

Figure 5 shows the loadings for the four descriptors that are most important in the classification of compounds. More potent compounds have high contributions from the descriptors QO11, HE and D2, while less potent compounds have a high contribution from the descriptor RTe^+^. Thus, the descriptors QO11, HE and D2 are responsible for the location of more potent compounds at the bottom of the graph. The descriptor RTe^+^ places less potent compounds in the upper part of the graph. Figure 5 also shows that the higher the contribution of the descriptor RTe^+^ in the second principal component, *i.e*., the higher the value of the maximum index of R/Sanderson electronegativity for a certain compound, the higher the score value will be, indicating that the compound is less potent than others. The other descriptors contribute to a lesser degree. For example, the descriptor HE has negative weight in PC2, demonstrating that the most potent compounds generally have higher values of this descriptor.

Costa *et al*. [40] showed that the presence of water changed the dihedral angle involved in the heme–artemisinin complex (C–Fe––O1–O2). Thus, this effect is believed to influence the process of molecular recognition between artemisinin and derivatives and heme in aqueous biological systems. The selection of the torsion angle D2 (O2–O1–Fe–N2) descriptor suggests that the action of drugs against malaria depends on electrophilic attack on the endoperoxide bond, particularly on the O1 atom. This result was confirmed by both an analysis of the *MEP* maps and by molecular docking as discussed previously.

### 2.5. HCA Results

**Figure 6:**
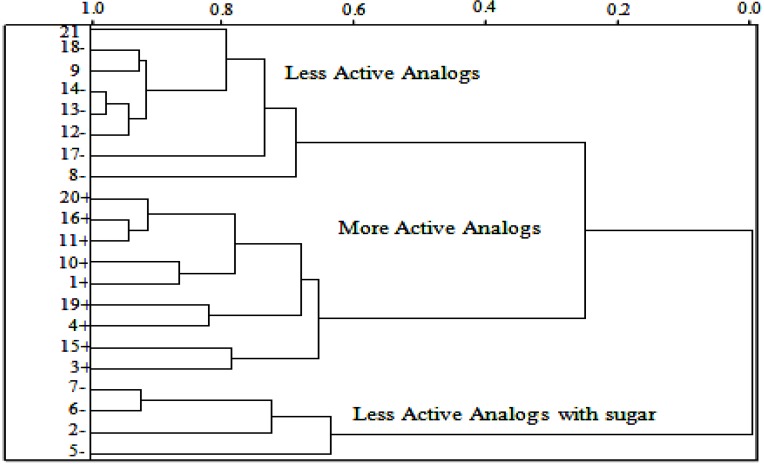
HCA dendrogram for artemisinin and derivatives with biological activity against W-2 strains of *P. falciparum.* Positive values indicate more potent analogs, and negative values indicate less active compounds.

The statistical analysis utilized in this study should group similar compounds into categories. The categories are represented by a two-dimensional diagram known as dendrogram that illustrates the fusions or divisions made at each successive stage of the analysis. Single samples (compounds) are represented by the branches on the bottom of the dendrogram. The similarity among the clusters is given by the length of their branches, so compounds presenting low similarity have long branches whereas compounds of high similarity have short branches. The HCA method classified the compounds into three classes (more active, less active and less active containing sugar) and was based on the Euclidean distance and the incremental method [50]. In the incremental linkage, the distance between two clusters is the maximum distance between a variable in one cluster and a variable in the other cluster. The descriptors employed to perform HCA were the same as those used for PCA, *i.e*., *HE*, *QO11*, *D2 (O2–O1–Fe–N2)* and RTe^+^. In the HCA technique, the distances between pairs of samples are computed and compared. Small distances imply that compounds are similar, while dissimilar samples will be separated by relatively large distances. The dendrogram in Figure 6 shows the HCA graphic as well as the compounds separated into three main classes. The scale of similarity varies from 0 for samples with no similarity to 1 for samples with identical similarity. By analyzing the dendrogram, some conclusions can be drawn even though the compounds present some structural diversity. HCA showed results similar to those obtained with PCA. The compounds are grouped according to their biological activities. The most potent compounds are **1**, **3**, **4**, **10**, **11**, **15**, **16**, **19** and **20**. The less potent compounds are grouped into two clusters, one of which contains compounds **8**, **9**, **12**, **13**, **14**, **17**, **18** and **21**, and the other cluster contains artemisinin derivatives that possess a sugar (2, 5, 6 and 7).

### 2.6. Partial Least Squares (PLS) and Principal Component Regression (PCR) Results

The statistical quality [51] of the PLS and PCR models was gauged by parameters such as correlation coefficient or squared correlation coefficient (R^2^), explained variance (R^2^_ajust_, *i.e*., adjusted R^2^), standard deviation (s), variance ratio (F), cross-validated correlation coefficient (*Q*^2^), standard error of validation (*SEV*), predicted residual error sum of squares (*PRESS*) and standard deviation of cross-validation (*S_PRESS_*) [52,53,54]. The best regression models were selected based on high values of R^2^, R^2^_ajust_, Q^2^ and F (a statistic of assessing the overall significance) and low values of s, *SEV*, *PRESS* and *S*_press_.

The calculated properties and the experimental activity values for the compounds studied (Table 5) were used to build the regression models. The models built using the PLS and PCR methods were based on three latent variables, 18 test compounds and 3 compounds (**2**, **12** and **13**) from the external validation set.

**Table 5:** Predicted PLS and PCR results and validation errors for log*RA* (experimental).

Compounds	Predicted		Validation Error		Experimental
	PLS	PCR	PLS	PCR	log*RA*
**1+**	0.2548	0.0902	0.2548	0.0902	0.0000
**2−** ^[a]^	−1.0163	−0.8805	−0.935	−0.7992	−0.0813
**3+**	−0.2855	−0.6548	−0.8392	−1.2085	0.5537
**4+**	0.2199	−0.1981	−0.1212	−0.5392	0.3411
**5−**	−2.1192	−1.7899	0.2812	0.6105	−2.4004
**6−**	−1.6837	−1.4214	0.0376	0.2999	−1.7213
**7−**	−1.8113	−1.5164	−0.1115	0.1834	−1.6998
**8−**	−0.1174	0.2199	−0.1111	0.2262	−0.0063
**9−**	0.0948	0.2284	0.1011	0.2347	−0.0063
**10+**	0.0342	−0.1872	−0.3833	−0.6047	0.4175
**11+**	0.4495	0.2485	0.4232	0.2222	0.0263
**12−** ^[a]^	−0.1670	0.1032	1.5524	1.8226	−1.7194
**13−** ^[a]^	−0.0920	0.1144	0.9807	1.1871	−1.0727
**14−**	−0.0583	0.1144	0.249	0.4217	−0.3073
**15+**	0.1452	−0.0974	−0.7151	−0.9577	0.8603
**16+**	0.3812	0.1289	0.0742	−0.1781	0.3070
**17−**	0.0203	0.4326	0.5917	1.004	−0.5714
**18−**	−0.0448	0.0392	0.2128	0.2968	−0.2576
**19+**	0.0888	−0.2535	−0.2654	−0.6077	0.3542
**20+**	0.3283	0.1684	0.3066	0.1467	0.0217
**21−**	−0.6913	−0.3463	0.0142	0.3592	−0.7055

^[a]^ Compounds from the external validation set.

The regression Equations obtained for PLS (Equation (3)) and PCR (Equation (4)) models that relate the descriptors and biological activity are the following:

*Log RA* = 0.520565HE - 0.151382QO11 - 0.158294RTe^+^ + 0.353200D2 (3)

*n* = 18, R^2^ = 0.9468, R^2^_ajust_ = 0.9354, s = 0.2211, F_(4,11)_ = 57.8889, *Q^2^* = 0.8566, *SEV* = 0.3202, *PRESS* = 0.6847, *S_PRESS_* = 0.0636.

*Log RA* = 0.304816HE - 0.258614 QO11 + 0.046881RTe^+^ + 0.340163D2 (4)

*n* = 18, R^2^ = 0.8488, R^2^_ajust_ = 0.8164, s = 0.3729, F_(4,11)_ = 18.2454, *Q^2^* = 0.7313, *SEV* = 0.4438, *PRESS* = 1.9476, *S_PRESS_* = 0.1073.

The results obtained with the PLS and PCR models were very close, with variation of R^2^ = ±0.098, R^2^_ajust_ = ±0.119, s = ±0.1518, F_(4,11)_ = ±39.6435, *Q^2^* = ±0.1253, *SEV* = ±0.1236, *PRESS* = ±1.2629 and *S_PRESS_* = ±0.0437 (between PLS and PCR). The quality of the PLS and PCR models can be demonstrated by comparing the measured and the predicted activities. The validation errors obtained by the leave-one-out cross-validation method are shown in Table 5. For the PLS, model only five compounds (**2**, **3**, **12**, **13** and **15**) had high validation errors, and the PCR model yielded five compounds (**3**, **12**, **13**, **15** and **17**) with high residual values. Our PLS and PCR models present the best fit for compounds with high activity because compounds with low activity showed high residuals values.

**Figure 7:**
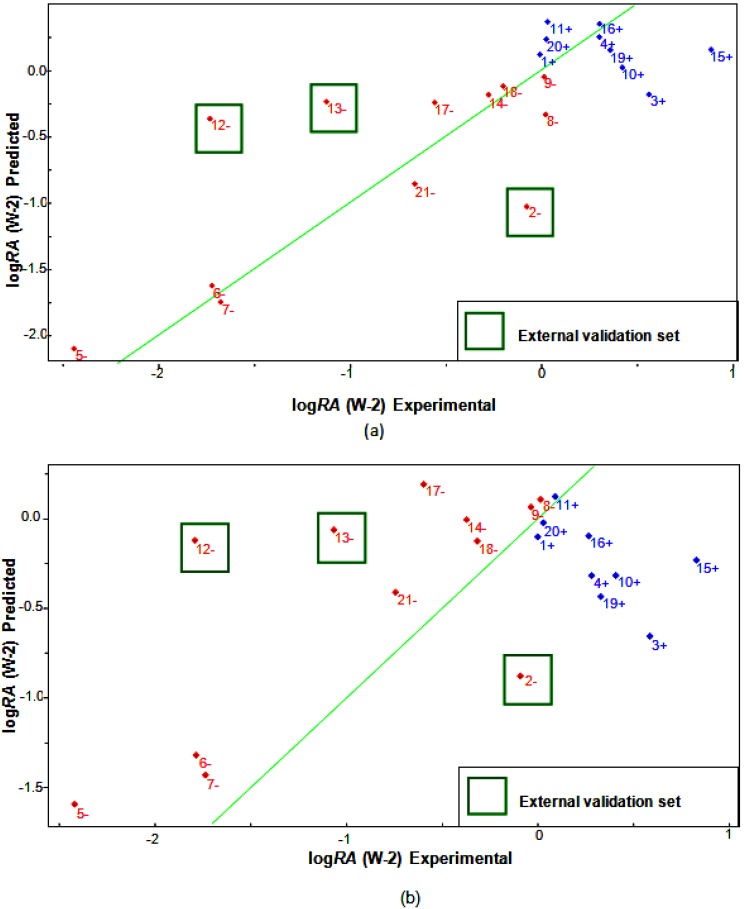
Plot of experimental versus predicted values for log*RA* modeled by (**a**) PLS and (**b**) PCR.

The measured versus predicted values using our PLS and PCR models are presented in (Figure 7a,b) respectively. The PLS and PCR plots identify compounds with higher activity (blue) and compounds with lower activity (red), including compounds from the external validation set. According to the PLS and PCR models, the four variables present different magnitudes of regression coefficients (in absolute value). The models reveal that compounds with high biological potency against *P*. *falciparum* have a combination of higher values of HE and D2 and lower values of QO11 and RTe^+^ for the PLS model, but for the PCR model, compounds have higher values of HE and D2, lower QO11 values and positive values for RTe^+^. The validation parameters support the fact that the models are efficient and hence satisfactory given the complexity of the antimalarial mechanisms and the small number of descriptors (four) selected to build the QSAR model.

The compounds of the set test were molded from the most stable structure of artemisinin, compound **1** of Figure 1, and constructed using GaussView 5.0 program, carrying the complete optimization of the geometry of each compound with the basis set of separated valence 6-31G** using the Hartree-Fock method as implemented in Gaussian 03 program. After obtain the most stable geometry of each compound was determined only selected descriptors in PCA and used in the construction of the QSAR (PLS and PCR) models, namely EH, QO11, RTe^+^ and D2, shown in Table 6.

**Figure 8:**
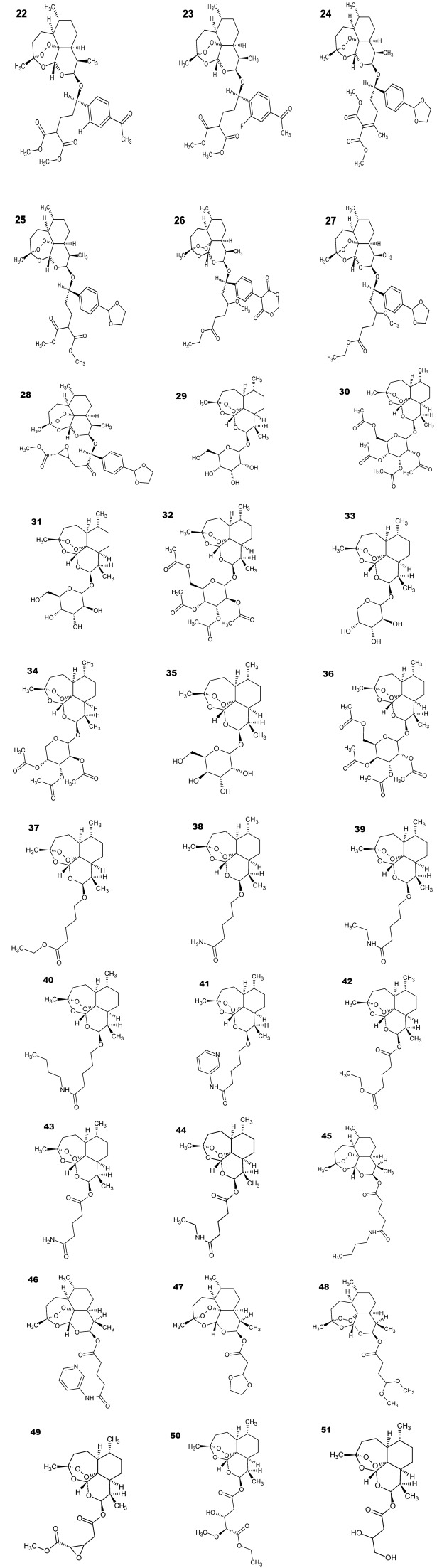
Compounds of the test set artemisinin derivatives with unknown antimalarial activity against *Plasmodium falciparum* type W-2.

The QSAR models (PLS and PCR) were built used to predict the unknown antimalarial activity of thirty new artemisinin derivatives shown in Figure 8, compounds **22**–**51**. Table 7 shows the results of the log*RA* by PCR and PLS models. According to Table 7 the PLS model showed that fifteen compounds of the test set (**22**, **23**, **27**, **30**, **32**, **34**, **36**–**40**, **44**, **45**, **47**, **51**) are predicted to be more active, they had values of log*RA* greater than zero (log*RA* > 0). However, the PCR model only nine compounds of all test sets (**23**, **25**, **37**, **38**, **43**, **46**, **48**–**50**) were predicted as most active, which showedvalues of log*RA* higher than zero (log*RA* > 0), a total of 24 compounds proposed as more active of thirty suggested compounds. However, compounds **23**, **37** and **38** were the ones that had values of log*RA* greater than zero (log*RA* > 0) in both models (PLS and PCR) with residues of prediction ranging from 0.028951 to −0.1351, suggesting that these new compounds in the two models (PLS and PCR) are more potent than artemisinin may be synthesized and tested for antimalarial activity.

**Table 6:** Molecular properties selected by analysis of main components of test set with antimalarial activity unknown.

Test Set	EH	QO11	RTe^+^	D2
**22**	−3.460	−0.663	0.076	7.585
**23**	−3.370	−0.664	0.077	141.065
**24**	−4.790	−0.556	0.069	130.453
**25**	−5.780	−0.675	0.077	98.153
**26**	−8.070	−0.603	0.076	−76.018
**27**	−4.650	−0.602	0.073	−4.170
**28**	−7.440	−0.575	0.066	−9.051
**29**	−15.920	−0.482	0.100	73.480
**30**	−4.470	−0.594	0.070	125.875
**31**	−15.240	−0.601	0.106	9.276
**32**	−4.500	−0.532	0.063	−37.529
**33**	−13.680	−0.578	0.126	−83.125
**34**	−4.550	−0.572	0.071	8.222
**35**	−13.620	−0.523	0.121	32.018
**36**	−4.280	−0.584	0.071	−27.718
**37**	−2.740	−0.650	0.105	152.098
**38**	−2.850	−0.673	0.081	101.819
**39**	−2.680	−0.603	0.068	−13.617
**40**	−3.290	−0.577	0.064	−65.438
**41**	−10.210	−0.615	0.122	10.190
**42**	−7.044	−0.557	0.062	−13.671
**43**	−7.841	−0.654	0.131	127.514
**44**	−2.910	−0.657	0.072	−25.670
**45**	−2.870	−0.670	0.069	−19.115
**46**	−7.020	−0.745	0.155	95.479
**47**	−4.240	−0.600	0.066	122.578
**48**	−8.120	−0.684	0.123	131.353
**49**	−8.350	−0.665	0.134	105.669
**50**	−5.676	−0.667	0.126	113.564
**51**	−3.640	−0.636	0.067	7.855

**Table 7:** Antimalarial activity predicted (log AR) by PCR and PLS models for the test set compounds and residues of prediction between models.

Test Set Compounds	Predicted (log*RA*)		Residues of Prediction (PLS−PCR)
	PLS	PCR	
**22**	0.28515	−0.08153	0.36669
**23**	0.16134	0.29643	−0.13510
**24**	−0.02669	−0.17894	0.15225
**25**	−0.13236	0.11823	−0.25059
**26**	−0.27509	−0.71065	0.43556
**27**	0.13050	−0.37989	0.51040
**28**	−0.25528	−0.60997	0.35469
**29**	−1.52738	−1.01052	−0.51686
**30**	0.02281	−0.04421	0.06701
**31**	−1.36654	−0.73626	−0.63028
**32**	0.18562	−0.72371	0.90933
**33**	−1.05361	−0.98490	−0.06871
**34**	0.13185	−0.44910	0.58095
**35**	−1.16288	−0.86252	−0.30036
**36**	0.20640	−0.49559	0.70198
**37**	0.23829	0.33770	−0.09941
**38**	0.27428	0.24533	0.02895
**39**	0.41609	−0.32859	0.74468
**40**	0.38362	−0.59287	0.97649
**41**	−0.66296	−0.46071	−0.20225
**42**	−0.19509	−0.67464	0.47955
**43**	−0.45107	0.10809	−0.55916
**44**	0.39618	−0.17717	0.57335
**45**	0.39508	−0.11549	0.51058
**46**	−0.30336	0.40099	−0.70434
**47**	0.05839	−0.02784	0.08623
**48**	−0.49407	0.20276	−0.69682
**49**	−0.50004	0.06913	−0.56917
**50**	−0.13357	0.19691	−0.33048
**51**	0.25967	−0.19372	0.45339

The most potent compounds have log*RA* ≥ 0; the less potent compounds have log*RA* < 0.

## 3. Experimental

### 3.1. Compounds Studied

Initially, 21 molecules were selected from the literature (Figure 1). Compounds **2**–**7** were proposed by Lin *et al*. [55], who found that the acetylation of dihydroartemisinin derivatives containing a sugar leads to similar or better activity than that of artemisinin. However, the deacetylation of sugars considerably reduces the antimalarial activity. Compounds **8**–**13** were chosen to examine the impact of the stereospecificity of the alkyl side chain on the biological properties and were proposed by Lin *et al*. [56] to obtain compounds with better biological activity than the antimalarials artelinic acid, artemisinin, artemether and arteether. However, the conversion of esters to their corresponding acids dramatically reduces their antimalarial activity. Compounds **14**–**21** were proposed by Lin *et al*. [57,58] because large substituents on the α-methylene carbon (*C) substantially increase the antimalarial activity of the compounds on the basis of electronic and steric effects and because the increased lipophilicity of artemisinin derivatives results in increased antimalarial activity. They were chosen for their *in vitro* bioactivity against the drug-resistant malarial strain *P. falciparum* (W-2 clone), which is chloroquine resistant but mefloquine sensitive. The numbering of the atoms used in this study is shown in Figure 1 (compound **1**—artemisinin). Because biological data were obtained from different sources, the logarithm of the *IC*_50_ value of artemisinin over the *IC*_50_ value of the compounds (logarithm of relative activity, log *RA*) was used to reduce inconsistencies caused by individual experimental environments:

log *RA* = log (IC_50_ of artemisini n / IC_50_ of an analog) (5)

where *IC*_50_ is the 50% inhibitory concentration. In this study, the following classification based on the antimalarial responses was adopted: compounds with log*RA* ≥ 0.00, ranging from 0.00000 to 0.86031, were assumed to be more potent analogs (1, 3, 4, 10, 11, 15, 16, 19 and 20), and those with log*RA* < 0.00, ranging from −0.00634 to −2.40049, were considered to be less potent analogs (**2**, **5**–**9**, **12**–**14**, **17**, **18** and **21**). Based on the relative activity (RA) values, compounds **3**, **4**, **10**, **15**, **16**, and **19** are 2–7 times more potent than artemisinin. Compound **15** is the most potent compound in the series studied.

### 3.2. Geometric Optimization and Descriptor Calculations

Molecular modeling started with the construction of the structure of artemisinin using GaussView 3.0 program [59], which was then optimized with different methods and basis sets—semiempirical (AM1, PM3 and ZINDO), ab initio/Hartree-Fock (HF/6-31G, HF/6-31G* and HF/6-31G**) and DFT (B3LYP/3-21G, B3LYP/3-21G* and B3LYP/3-21G**). These calculations were performed to find the method and basis sets with the best fit between the computational time and accuracy of the information compared to the experimental data [39]. After initial determination and structural optimization of artemisinin, the theoretical geometrical parameters of artemisinin in the region of the 1,2,13-trioxane ring (bond length, bond angle and torsion angle of the atoms that form this ring) were determined with the aim of evaluating the quality of the molecular wave function comparing the theoretical geometrical parameters with the experimental data (Table 1). The experimental structure of artemisinin was taken from the Cambridge Structural Database CSD, with REFCODES: QNGHSU10, crystallographic R factor 3.6 [60]. All the other structures (Figure 7) were built with the optimized structure of artemisinin using the Gaussian 03 program [61] with the Hartree-Fock (HF) method and 6-31G** basis set. After the structures were determined in three dimensions, various descriptors for each molecule of the set studied were calculated. They represent different sources of chemical information (features) regarding the molecules and include geometric, electronic, quantum-chemical, physical-chemical and topological descriptors, among others. They are important for the quantitative description of molecular structure and to finding appropriate predictive models [62]. The computation of the descriptors was performed employing the following software: Gaussian 03 program, [61] e-Dragon [63,64], Autodock 4.0 [65], Molekel [66] and HyperChem 6.02 [67]. With aid of the e-Dragon program, 1666 calculated descriptors were divided into the following 20 classes: 48 constitutional descriptors; 47 descriptors of quantity and trajectory; 47 information indexes; 107 adjacency indexes; 21 topological charge indexes; 41 molecular Radic profiles; 150 RDF descriptors; 154 functional groups; 14 charge descriptors; 33 connectivity indexes; 96 2-D autocorrelations, 64 Burden eigenvalues; 44 indexes based on eigenvalues; 74 geometric descriptors; 160 MORSE-3D; 120 fragments centered in the atom; 31 molecular property descriptors; 119 topological indexes; 99 WHIM descriptors; and 197 Getaway descriptors. Other descriptors such as the following were obtained:

(a) *MOLECULAR DOCKING* descriptors*:* These were calculated to better represent the interaction between the drug and receptor with the aid of the AutoDock 4.0 program. The following 17 molecular docking descriptors were included in the data matrix: binding energy (BE); partition function (Q), Gibbs free energy (G), internal energy (U), electrostatic energy (EE); bond length (Fe–O1, Fe–O2, Fe–O13 and Fe–O11), bond angle (O2–O1–Fe, O1–O2–Fe, C4–O1–Fe and C5a–O1–Fe); and dihedral angle (O2–O1–Fe–N1, O2–O1–Fe–N2, O2–O1–Fe–N3 and O2–O1–Fe–N4).
(b) *QUANTUM CHEMICAL* descriptors*:* In our study, we calculated the following 25 quantum-chemical descriptors: total energy (ET), energy of the highest occupied molecular orbital (HOMO), a level below the energy of the highest occupied molecular orbital (HOMO-1), lowest unoccupied molecular orbital energy (LUMO), a level above the energy of the lowest unoccupied molecular orbital (LUMO + 1), difference in energy between HOMO and LUMO (GAP = HOMO-LUMO), Mulliken electronegativity (χ), molecular hardness (η), molecular softness (1/η), and charge on the atom n (where *n* = 1, 2, 3, 4, 5, 5a, 6, 7, 8, 8a, 9, 10, 11, 12, 12a, 13). The atomic charges used in this study were obtained with the key word POP = CHELPG using the electrostatic potential [68]. With this strategy, it was possible to obtain the best potential quantum molecular series of points defined around the molecule, and atomic charges offer the general advantage of being physically more satisfactory than Mulliken charges [69].
(c) *Descriptors related to quantitative properties of chemical structure and biological activity*: In our data matrix, QSAR descriptors were included, *i.e.*, total surface area (TSA), molecular volume (MV), molar refractivity (MR), molar polarizability (MP), coefficient of lipophilicity (logP), molecular mass (MM) and hydration energy (HE) according to the HyperChem 6.02 program. The molecular descriptors were selected to provide valuable information about the influence of electronic, steric, hydrophilic and hydrophobic features on the antimalarial activity of artemisinins.

### 3.3. Interaction between Artemisinins and Heme

The interaction between the ligands (artemisinins) and the receptor (heme) was studied with molecular docking to determine the best geometry for the complex formed between these two molecules. The geometry of artemisinin and its derivatives (ligands) was designed with HF/6-31G**, whereas the geometry of heme (receptor) was obtained from the 1A6M structure in the RCSB protein data bank (PDB) from Vojtechovsky *et al.* [70]. The arrangement in the docking calculation took into account the presence of the proximal histidine residue under the plane of the porphyrin ring. This histidine moiety is, as usual, perpendicularly coordinated to Fe^2+^ through the sp^2^ nitrogen atom of its imidazole ring. Such an arrangement allows the Fe^2+^ to attain a nearly octahedral hexacoordinated arrangement after binding to the artemisinin molecule [14]. The orientation of the ligand was set just above the plane of the heme. Then, for each ligand/receptor interaction, 100 conformations were calculated, and the most probable one was determined based on the lowest energies of interaction. Automated docking calculations were performed to develop possible conformations for the complex employing the Lamarckian Genetic Algorithm implemented in the package Autodock 4.0. This program starts the docking by displaying the ligand in an arbitrary conformation and position and looking for favorable dockings with the receptor using both simulating annealing and genetic algorithms. AutoDock uses a random number generator to create new poses for the ligand during its search and estimates the free energy of binding of a ligand to its target. The resulting conformations were ranked in order of increasing binding energy of the lowest binding energy conformation in each cluster.

### 3.4. Molecular Electrostatic Potential Maps

An important concept explored in this research was the correlation of the structure activity of the species studied here through the characteristics of the electrostatic potential in the region of the 1,2,13-trioxane ring because in the literature, artemisinin and derivatives with antimalarial activities present similar patterns in their *MEP* maps [36,37,71]. Such a method enables the use of a qualitative analysis to locate reactive sites in a molecule and determine the roles played by both the electronic and steric (size/shape) effects on its potency. It is worthwhile to note that the visualization of *MEP* maps provides qualitative information on molecules, such as the behavior of the interaction between a ligand and a receptor. The MEP at a given point (x, y, z) in the vicinity of a molecule is defined in terms of the interaction energy between the electrical charge generated from the molecules electrons and nuclei and a positive test charge (a proton) located at ***r***. For the studied compounds, the V(*r*) values were calculated by Equation (6) as described previously (see [72]):

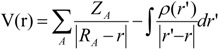
(6)
where Z_A_ is the charge of nucleus A, located at R_A_, ρ(r') is the electronic density function of the molecule, and r' is the dummy integration variable. The *MEP* maps for artemisinin and its derivatives were computed from the atomic charge at the HF/6-31G** level using the Gaussian 03 program, and the results are displayed with Molekel software.

### 3.5. Variable Selection and Model Building

After the determination of all molecular descriptors, it was possible to construct a data matrix to develop step multivariate analysis. Before we began the multivariate analysis, it was necessary to make the autoscale or standardizing data matrix X = (n, m) consisting of 21 lines (the compounds studied) and 1,733 columns (in this case, the calculated descriptors for each molecule), where n is the number of studied compounds and m is the number of variables. The aim of using the standardizing matrix is to give each variable equal weight in mathematical terms, so each variable was centered on the mean and scaled to unit variance. To reduce the data set, variables were selected based on the analysis of the correlation matrix between variables (descriptors) and the logarithm of the relative activity (log*RA*). Those with small or no correlation (under the 0.30 correlation value cutoff) were discarded, except for QSAR and quantum chemical descriptors, resulting in only 230 descriptors remaining from the initial set of 1,733 descriptors. After this data compression, two complementary methods for exploratory data analysis were employed (PCA and HCA) to study intersample and intervariable relationships and to select the properties that contribute the most to the classification of the compounds into two groups. One group contained more potent analogs and the other less potent analogs. PCA was employed to reduce the dimensionality of the data, find descriptors that could be useful in characterizing the behavior of the compounds acting against malaria and look for natural clustering in the data and outlier samples. While performing PCA, several attempts to obtain a good classification of the compounds were made. At each attempt, the score and loading plots were analyzed based on the variables employed in the analysis. The score plot gives information about the compounds (similarity and differences). The loading plot gives information about the variables (how they are connected to each other and which best describe the variance in the original data). The descriptors selected by PCA were used to perform HCA, PLS and PCR. The objective of HCA was to present the compounds distributed in natural groups and the results confirm the PCA results. Thus, several approaches were attempted to establish links between samples/cluster. All of them were of an agglomerative type because each sample was first defined as its own cluster, and then others were grouped together to form new clusters until all the samples were part of a single cluster.

The QSAR models for the artemisinin compounds studied were constructed by the PCR and PLS methods based on the autoscaled data and the leave-one-out crossvalidation procedure [28,29]. The final purpose of the multivariate analysis (PLS and PCR) was the construction of a mathematical model that can be used to predict antimalarial activity. The samples selected to compose the external validation set were **2**, **12** and **13**. The statistical parameters used to assess the quality of the models were the Prediction Residual Error Sum of Squares (*PRESS*), Equation (7), the Standard Error of Validation (*SEV*), Equation (8), the total variance explained, *R*^2^ (correlation between the estimated values predicted by the model built with the full data set and actual values of *y*), *Q*^2^ (the cross-validated correlation coefficient) and *S_PRESS_* (standard deviation of cross-validation) given by Equations (9)–(11), respectively [28,29,73,74,75]:

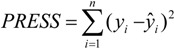
(7)

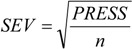
(8)

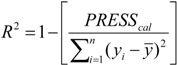
(9)

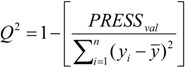
(10)

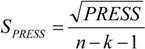
(11)


In Equations (7) and (8), *n* is the number of compounds used for the calibration or validation model, *y_i_* is the experimental value of the physicochemical property for the sample and *ŷ_i_* is the value predicted by a calibration or validation model. In Equations (9) and (10), *PRESS_cal_* is the Calibration Prediction Error Sum of Squares and *PRESS_val_* is the Validation Prediction Error Sum of Squares. Both *PRESS_cal_* and *PRESS_val_* are evaluated from Equation (7) by changing *ŷ_i_* for a calibration or validation model. The values of explained variance (R^2^_A_, *i.e*., adjusted R^2^), standard deviation (s) and F (Fisher test) were determined. The multivariate data analyses (PCA, HCA, PLS and PCR) were performed by employing the Pirouette 3.01 software [50].

## 4. Conclusions

The HF method and the 6-31G** basis set revealed themselves to be adequate to optimize the structures of artemisinin and derivatives for consequent study. The molecular docking studies reinforced the idea that the Fe^2+^ ion from heme preferentially binds the O1 atom from artemisinins rather than the O2 atom and that such a preference may be due to a greater steric hindrance at O2 than O1 and a more negative charge on the latter atom. Both factors are essential for intermolecular approach. MEP maps characterize the region of the 1,2,13-trioxane ring in artemisinin and derivatives as a region of negative electrostatic potential, and the use of MEP maps identified key structural features necessary for antimalarial activity. Investigation of the interaction with the molecular receptor (heme) showed that the presence of a red surface near the 1,2,13-trioxane ring suggested that these compounds have a reactive site for electrophilic attack. This attack preferentially occurs through the endoperoxide linkage. The predictive classification models for artemisinin derivatives were obtained with a set of molecular descriptors selected by chemometric approaches. PCA and HCA methods classified the studied compounds into groups according to their degree of antimalarial activity against *P. falciparum* (W-2 clone). The descriptors hydration energy (HE), charge on oxygen atom of O11 (QO11), torsion angle O1–O2–Fe–N2 (D2) and maximum rate of R/Sanderson Electronegativity (RTe^+^) were responsible for distinguishing compounds with higher and lower antimalarial activity. The molecular features represented by these descriptors are in good agreement with previous SAR analysis performed on artemisinin derivatives. The combination of these structural attributes is believed to govern the antimalarial effects of the compounds studied in this work. The PLS and PCR models obtained here showed not only statistical significance but also predictive ability. Through this strategy and our findings, useful information was obtained that could be of use in experimental syntheses and biological evaluation to understand the molecular and structural requirements for designing new ligands to be used as antimalarial agents.
